# Macromolecular dimensions of a synthetic polyelectrolyte as a factor in its interactions with protein and cells: desirability for longer chains

**DOI:** 10.1039/d5tb02163d

**Published:** 2025-11-27

**Authors:** Raman Hlushko, Alexander Marin, Ananda Chowdhury, Alexander K. Andrianov

**Affiliations:** a Institute for Bioscience and Biotechnology Research, University of Maryland Rockville MD 20850 USA aandrianov@umd.edu

## Abstract

Understanding the mechanism of interactions between synthetic polyelectrolytes and ionic matter, which are ubiquitous in living systems, such as proteins and cells, is a fundamental challenge and an important requirement for their clinical development as biomaterials and drug delivery systems. In contrast to small molecules or proteins, in which an active center or epitope largely defines the binding pattern, ionic polymers utilize a plurality of repeat units, which are capable of only weak interactions with the target individually. Although it can be expected that the effects of the chain length and cooperativity play important roles in such interactions, these effects are often overlooked in practical research. Nevertheless, preclinical experience demonstrates the existence of an activity-molar mass relationship in polyelectrolytes. Here, we focus on studying the *in vitro* interactions of a clinical-grade macromolecule, poly[di(carboxylatophenoxy)phosphazene] (PCPP), for which such a relationship has already been established *in vivo*. We found that polymers of various molar masses show different *in vitro* avidities to a model antigenic protein, lysozyme, with longer PCPP chains displaying lower dissociation constants and reduced entropic penalties. Higher-molar-mass polymers result in less compact complex morphologies, in which the protein is more easily accessed by the antibody. The trend of the greater *in vitro* activation of engineered immune cells with longer polymer chains is also observed. Results suggest that morphological and entropic benefits provided by higher-molar-mass polymers are critical in explaining previously observed *in vivo* trends, and these aspects should be prioritized in designing next-generation macromolecular immunoadjuvants.

## Abstract 2

Longer-chain polyelectrolyte immunostimulants are favoured in interactions with proteins and cells. Lower protein-complexation entropy penalty, superior antigen presentation and immune cell activation explain their higher *in vivo* potency.

## Graphical abstract

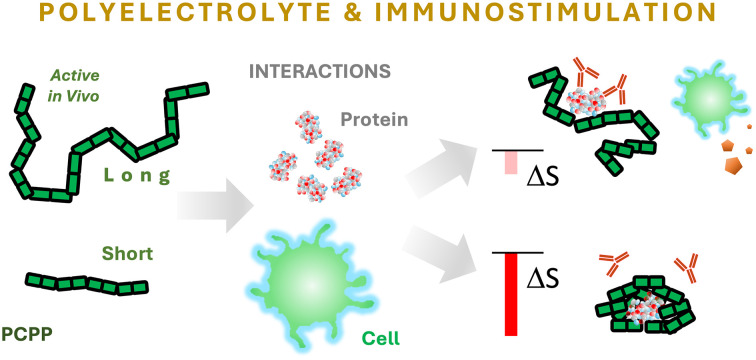

## 1. Introduction

Synthetic polyelectrolytes offer invigorating solutions for the development of multifunctional drug delivery systems and advanced biomaterials.^1–7^ Their ability to spontaneously self-assemble with ionic counterparts has inspired numerous studies on the behavior of ionic macromolecules in solutions and on the mechanisms of intermolecular complexation and multilayer polyelectrolyte assembly.^8–13^ An in-depth understanding of the way these macromolecules interact with proteins and cells (ionic matters), which are ubiquitous in nature, is especially important. Although this is quite challenging, it is a prerequisite for the successful advancement of ionic polymers into preclinical and clinical settings.^14,15^

Polyelectrolyte complexation, including the formation of complexes with proteins, is governed by a number of fundamental physicochemical parameters, mainly by the choice of ionic pairs and environmental conditions.^16^ Thermodynamically, polyelectrolyte interactions are dominated by favorable entropic contributions arising from the release of counterions; however, the free energy of complex formation is important for weak polyelectrolytes.^17^ Reactions of polyelectrolytes often involve mechanisms of cooperativity and associative charging. For example, a highly ionized polyelectrolyte can promote the charging of its partner chain even under environmental conditions, which does not favor the dissociation of the latter.^17,18^

Interactions of polyelectrolytes with proteins, which are amphoteric in nature, are characterized by some unique phenomena. While complex formation between a polymer and a protein, carrying net charges of the opposite sign, is the subject of most investigations, interactions on the “wrong side” of the isoelectric point have also been reported.^19–22^ The binding of a polyelectrolyte and a protein with both carrying like-charges becomes possible due to an uneven distribution of charges on the protein surface and the flexibility of the polymer chain that can access those patches with sufficient efficiency.^19–22^ Macromolecular topology is yet another important parameter, which affects the way polyelectrolytes form complexes with proteins. In particular, polymers with complex star-shaped macromolecular architectures are reported to be less capable of protein binding than their linear counterparts.^23^

An in-depth understanding of polyelectrolyte-protein complexation is also a prerequisite for designing effective systems for intracellular delivery applications. Interactions of ionic macromolecular carriers with membrane proteins can facilitate the uptake of therapeutic agents by cancer cells or stimulate immune-competent cells required for the effective performance of vaccines.^14,24^ A relatively new strategy that utilizes ionic polymers and their avidity to proteins concerns cell surface engineering. The modification of the cell surface with polyelectrolytes has recently been brought under development as a versatile platform that allows the active modulation of cellular functions and creation of more realistic tissue structures.^3^

In studying interactions of polyelectrolytes with proteins and cells, one of the factors that is frequently overlooked is the length of a polymer chain. Nevertheless, this fundamental parameter has some significant practical implications. For instance, a member of a synthetic polyelectrolyte family – poly[di(carboxylatophenoxy)phosphazene] or PCPP, which has been advanced into clinical trials as part of vaccine formulations – has demonstrated a strong dependence of activity on its molar mass.^24^ An increase in its chain length is reported to boost the *in vivo* immunostimulatory potency of this macromolecule.^25^ The mechanism of such a phenomenon has not been elucidated, neither on the molecular level nor on the immunological pathway level. Gaining insights into the underlying reasons for such behavior may identify structural design parameters critical for the development of next-generation polymers displaying superior immunoadjuvant activity. This knowledge may also improve the persistence of immune responses, as polyphosphazene adjuvants are expected to lose activity as they undergo hydrolytic degradation with the formation of shorter-chain fragments.

Here, we synthesized and characterized six PCPP samples with mass average molar masses ranging from 35 to 1400 kDa. While studying their interactions with a model antigenic protein, hen egg lysozyme, we found that the thermodynamic patterns of binding displayed by shorter chains are characterized by higher dissociation constants and pronounced entropy penalties. Investigation of complex aggregation profiles by dynamic light scattering (DLS) and lysozyme accessibility in complexes by antibody binding suggested a more compact morphology for complexes formed by low-molar-mass polymers. Altogether, longer polymer chains showed higher avidity to lysozymes and a superior ability to display antigenic protein, which can provide important clues to the understanding of PCPP behavior *in vivo*. Furthermore, in experiments with engineered immune cells *in vitro*, high-molar-mass PCPP samples were able to induce higher activation levels, which can be potentially explained by their greater avidity to proteins on the cell surface.

## 2. Materials and methods

### 2.1. Materials

Propylparaben, diglyme (anhydrous, 99.5%), potassium hydroxide (Sigma-Aldrich, St. Louis, MO), propylparaben sodium, NF (Spectrum Chemical Mfg. Corp., New Brunswick, NJ), hen egg lysozyme (BioUltra, lyophilized powder, ≥98%), ethanol (200 proof, anhydrous) (The Warner-Graham Company, Cockeysville, MD) and phosphate buffered saline (PBS, pH 7.4) (Thermo Fisher Scientific, Waltham, MA) were used as received.

### 2.2. Synthesis of PCPP samples with variable molar masses

The synthesis of PCPP molar mass samples was carried out by reacting polydichlorophosphazene (PDCP) with propylparaben (*n*-propyl ester of *p*-hydroxybenzoic acid) and subsequent hydrolysis of ester functionalities.^26,27^ To achieve variations in the molar mass, propylparaben was utilized in quantities and under conditions that allowed for a controlled degradation of the partially substituted polymer in the reaction. For example, a high-molar-mass PCPP H2 sample was synthesized using a propylparaben in a reaction with PDCP in an equivalent of 22 as follows. Propylparaben (40 g, 0.22 mol) was dispersed in 22 mL of diglyme and heated in a three-neck round-bottom flask at 110 °C under stirring until fully dissolved. Sodium propylparaben (44.8 g, 0.22 mol) was then added, and the mixture was stirred until complete dissolution. The solution was subsequently diluted with 90 mL of diglyme under continuous stirring. Then, a solution of PDCP (1.35 g, 0.02 mol of the repeating unit) in diglyme (30 mL) was added dropwise to the reaction mixture using an addition funnel under a nitrogen atmosphere with constant stirring. The reaction was maintained at 110 °C for 2 h and then cooled to 95 °C, and aqueous potassium hydroxide (13 M, 70 mL) was added slowly to the mixture. The resulting precipitate was dissolved in deionized water, precipitated by the addition of sodium chloride, redissolved in deionized water and precipitated again using ethanol. Other PCPP molar mass samples were synthesized similarly using the following propylparaben equivalents: H1 – 18 : 1, M2 – 5 : 1, M1 – 4.5 : 1, L2 – 3 : 1, L1 – 2.7 : 1.

### 2.3. Size-exclusion chromatography with a multiangle light scattering (SEC-MALS)

Size-exclusion chromatography with ultraviolet (UV), refractive index (RI), and multiangle light scattering (MALS) detectors was used to determine the mass average and number average molar masses of PCPP samples. The SEC-MALS equipment consisted of a Vanquish Flex system (Thermo Fisher Scientific Inc., Waltham, MA, USA) equipped with refractive index (OptiLab T-rEX, Wyatt Technologies, Santa Barbara, CA, USA), multiangle laser light scattering (MALS, Dawn HELEOS-II, *λ* = 658 nm, Wyatt Technologies, Santa Barbara, CA, USA) and dynamic light scattering (DLS, DynaPro NanoStar, Wyatt Technologies, Santa Barbara, CA, USA) detectors. Data acquisition and processing were performed using ASTRA software (Wyatt Technologies, Santa Barbara, CA, USA). Two SEC columns connected in series were employed for the analysis: TSKgel GMPW (7.5 mm × 30 cm, 17 µm, TOSOH Bioscience LLC, Japan) and TSKgel G3000PW (7.8 mm × 30 cm, 7 µm, TOSOH Bioscience LLC, Japan). The samples were dissolved in PBS (pH 7.4) with a concentration of 1.0 mg mL^−1^ and filtered through a 0.22-µm nylon syringe filter prior to analysis. PBS (pH 7.4) was filtered through a 0.1-µm PVDF filter and was used as an eluent. The following SEC conditions were used for each run: 1.0 mg mL^−1^ PCPP, a 50 µL injection volume, a 0.5 mL min^−1^ flow rate and a 25 °C column temperature.

### 2.4. Isothermal titration calorimetry (ITC)

The calorimetric titration was performed using a Nano ITC SV instrument (TA Instruments, Waters, New Castle, DE, USA) in an aqueous solution (50 mM phosphate buffer, pH 7.5). The polymer solution (0.125 mg mL^−1^) was placed into an isothermal chamber, and a lysozyme solution (2.5 mg mL^−1^) was used as a titrant. Thirty-two 8-µL injections were performed from a 250-µL syringe rotating at 36.7 rad s^−1^ with a 300-s delay between each injection. A heat release curve (microjoules per second *versus* seconds), generated by each injection, was processed using NanoAnalyze software, version 3.12.5 (TA Instruments, Waters, New Castle, DE, USA) to yield the heat associated with each injection. The above software was also used for data analysis, and a single set of identical sites (SSIS) binding model was employed to calculate the following thermodynamic parameters: binding constant (*K*_d_), reaction stoichiometry (*n*), enthalpy (Δ*H*), and entropy (Δ*S*) changes.

### 2.5. Analysis of protein-antibody binding in lysozyme-PCPP complexes

The accessibility of lysozyme in complexes formed by L1 and H1 polymers was tested using an enzyme-linked immunosorbent assay (ELISA) according to the methodology described previously.^28,29^ A 96-well plate was coated overnight (4 °C) with a solution (100 ng mL^−1^, carbonate buffer, pH 9.2, 100 µL per well) of the rabbit anti-chicken egg lysozyme antibody (Rockland Immunochemicals, Gilbertsville, PA). The coating solution was then removed, and the wells were washed three times with PBS (pH 7.4) and then treated with a blocking buffer (1% BSA/0.05% Tween-20 in PBS, 300 µL per well) for 1 h at 37 °C. The blocking buffer was removed by rinsing the wells four times with a washing buffer (0.05% Tween-20 in PBS). Complexes of two stoichiometries were prepared – (a) 0.096 mg mL^−1^ lysozyme with 0.72 mg mL^−1^ PCPP (1.5 : 1 lysozyme/–COOH, mmol eq.^−1^) and (b) 0.08 mg mL^−1^ lysozyme with 0.368 mg mL^−1^ PCPP (2.5 : 1 lysozyme/–COOH, mmol eq.^−1^) – and serially diluted with PBS (pH 7.4) for the analysis. Each sample (50 µL) was reconstituted in the equivalent volume of a blocking buffer (2× the concentration) and placed in the well. The plate was incubated at 37 °C for 1 h, and then, the wells were rinsed with a washing buffer. Next, the peroxidase-conjugated rabbit antichicken egg lysozyme antibody (Rockland Immunochemicals, Gilbertsville, PA) in a blocking buffer (500 ng mL^−1^) was added (100 µL per well), the plate was incubated at ambient temperature for 1 h, and the wells were rinsed with a washing buffer. To develop a colorimetric reaction, the 3,3′,5,5′-tetramethylbenzidine (TMB) substrate (1-Step Ultra® TMB-ELISA, Thermo Scientific, Rockford, IL) was added (100 µL per well). The plate was left for 20 min at room temperature, and the reaction was stopped by adding sulfuric acid (1 M, 50 µL per well). The absorbance was measured at 450 nm using a Multiskan Spectrum Reader (Thermo Fisher Scientific, Waltham, MA). Each experiment was performed in triplicate.

### 2.6. Interactions of PCPP with engineered immune cells

*In vitro* evaluation was conducted using engineered murine macrophages – RAW BLUE cells (InvivoGen, San Diego, CA) featuring an NF-κB/AP-1-inducible secreted embryonic alkaline phosphatase (SEAP) reporter gene. Cells were maintained in the culture in Dulbecco's modified Eagle medium containing glucose and l-glutamine (Thermo-Fisher Scientific, Grand Island, NY) supplemented with 10% fetal bovine serum (10%), penicillin streptomycin (1%) (Thermo-Fisher Scientific, Grand Island, NY) and normocin (100 µg mL^−1^) (InvivoGen, San Diego, CA). PCPP samples of various molar masses and concentrations in culture were added to an equal volume of RAW BLUE cells in 96-well plates (120 000 cells per well) and incubated at 37 °C in carbon dioxide (5%) for 20 h. The extent of the RAW BLUE cell activation was assessed spectrophotometrically by the conversion of the SEAP substrate p-nitrophenylphosphate (Millipore Sigma, St. Louis, MO). The culture supernatant was added to the reagent in a one-to-ten ratio by volume. The absorbance (405 nm) was read using a ThermoScientific SpectraMax plate reader (Molecular Devices, San Jose, CA). Each experiment was performed in triplicate.

## 3. Results and discussion

Binding and effective presentation of immunogenic proteins is one of the key prerequisites of contemporary vaccine delivery systems and immunoadjuvants.^30–33^ This enabling functionality is also fundamental to the adjuvant effect of polyphosphazene macromolecules *in vivo*.^24,34,35^ However, the existence of a link between the loss of biological activity with the decrease in the molar mass observed in animal studies and the ability of PCPP to bind proteins is not yet established, and the potential mechanism remains obscure. Searching for keys to understand the underlying processes can facilitate the development of next-generation polyphosphazene immunoadjuvants.

### 3.1. Synthesis of polymers with variable chain lengths using a modulated *in situ* degradation approach

**Scheme 1:**
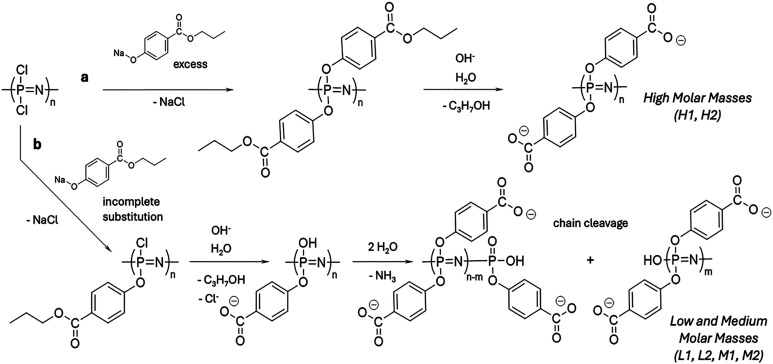
Synthesis of PCPP with varying molar masses utilizing (a) complete substitution of chlorine atoms on the polymer and (b) their controlled incomplete substitution, which results in the production of weak links and polymer chain cleavage.

Synthesis of PCPP macromolecules with variable chain lengths was achieved *via* the controlled degradation of a polymer in a production process. The synthetic pathway to PCPP utilized a ring-opening polymerization process with subsequent macromolecular substitution and deprotection reactions (Scheme 1a). This typically results in polymers with molar masses of approximately 1000 kDa.^26^ Macromolecules with shorter chain lengths were synthesized utilizing an incomplete substitution process, which yielded polymers containing residual chlorine atoms in the substitution step. These ‘weak links’ were then hydrolyzed in the deprotection step, resulting in the cleavage of the backbone and the formation of shorter polymer chains (Scheme 1b). Therefore, a reaction, in which propyl paraben was used in amounts that were not sufficient for the completion of the substitution process, produced the polymer with a lower molar mass. Accordingly, a greater deficiency of the reagent in the reaction mixture led to a larger in-process polymer degradation and a lower resulting molar mass. For each polymer in this study, the required reagent ratios were found empirically using the trial-and-error approach.

Using the procedures described above, six PCPP samples were synthesized and characterized (Table 1 and Fig. S1). They were grouped according to mass average molar masses (*M*_w_), determined by the SEC-MALS method: L1 and L2 (low–below 100 kDa), M1 and M2 (medium–between 100 and 1000 kDa) and H1 and H2 (high–above 1000 kDa). DLS characterization results were in agreement with SEC-MALS data on the chain length hierarchy of the synthesized samples (Table 1).

**Table 1:** Physicochemical characteristics of PCPP samples

N	Sample	SEC-MALS			DLS	
		*M*_n_ (kDa)	*M*_w_ (kDa)	*Đ*	*D*_*z*_ (nm)	pdi
1	L1	30	35	1.2	14	0.4
2	L2	40	50	1.4	20	0.4
3	M1	120	160	1.4	25	0.2
4	M2	240	280	1.2	43	0.3
5	H1	810	1100	1.4	57	0.4
6	H2	1100	1400	1.2	63	0.3

### 3.2. Higher-molar-mass polymers are capable of stronger protein binding, and their self-assembly is less penalized by entropy losses

**Fig. 1:**
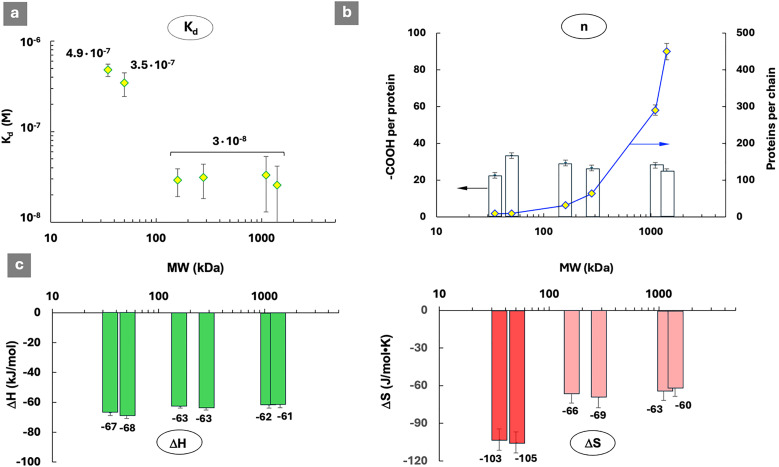
Isothermal titration calorimetry (ITC) results describing interactions between lysozyme and PCPP samples: (a) dissociation constants of the resulting complexes, (b) their stoichiometry, as described by the number of carboxylic acid groups per protein and proteins per PCPP chain, and (c) thermodynamic patterns of interactions (Δ*H*–enthalpy change, Δ*S*–entropy change; favorable contributions are shown in green, unfavorable in red, 0.125 mg mL^−1^ PCPP (analyte), 2.5 mg mL^−1^ protein (titrant), 50 mM phosphate buffer, pH 7.5).

The protein-binding properties of PCPP were studied using a hen egg lysozyme – a model vaccine antigen,^29,36,37^ which has been proven to work with PCPP *in vivo*.^38^ The results of isothermal titration calorimetry (ITC) analysis of interactions between PCPP and lysozyme are presented in Fig. 1 and Fig. S2–S7, SI. As seen from the comparison of dissociation constants, the avidity of PCPP to the protein is practically similar for mid- and high-molar-mass polymers, but lower for shorter-chain macromolecules – L1 and L2 (Fig. 1a). As expected, the number of protein molecules associated with a single polymer chain increases steadily as the molar mass of the polymer increases (Fig. 1b). The results indicate that it takes approximately 25–30 carboxylic acid groups of PCPP (12–15 repeat units) to bind a single lysozyme molecule. This number appears to be practically independent of the overall length of the polymer chain. The difference in the dissociation constant, which constitutes an increase of approximately one order of magnitude for shorter macromolecules, can be better understood from the comparison of thermodynamic patterns of interactions (Fig. 1c). As seen from the figure, in all cases, lysozyme–PCPP interactions are enthalpy-driven, which is consistent with processes resulting from electrostatic or hydrogen bonding between complementary functional groups of the synthetic macromolecule and those on the protein surface.^39^ This is generally consistent with interactions of negatively charged polymers and proteins with high isoelectric points.^40,41^ However, the observed gains are not significantly different across the whole molar mass range to explain an increase in the dissociation constants of complexes formed by L1 and L2.

In contrast to enthalpy changes, a significant difference is detected in entropy loss, with a larger penalty found in the case of both low-molar-mass macromolecules (Fig. 1c). The negative value of entropy change can be generally expected due to spatial constraints and compactization caused by complexation, but this effect can be at least partially compensated by the release of counterions and water molecules.^39,42^ The overall compactness of protein-polymer complexes and their water contents are determined by a number of fundamental parameters, such as chain flexibility and protein isoelectric point, and can vary in a broad range.^9^ Therefore, the observed differences in entropy changes can be useful to review in the context of the mechanism of complex formation, its morphology and the effect of chain length on these parameters.

### 3.3. Polymers with longer chain lengths display less compact morphologies

**Fig. 2:**
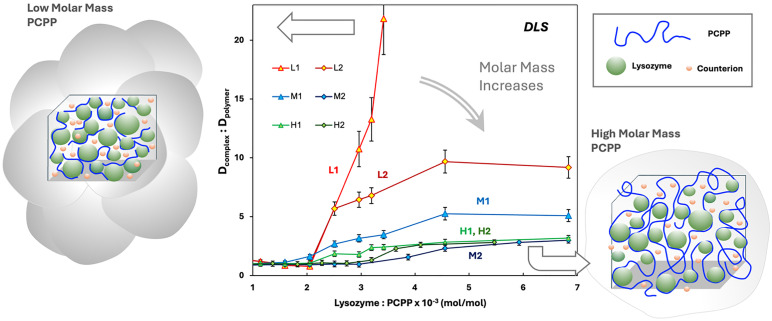
DLS titration of PCPP of variable molar masses with lysozyme. The results are presented as the ratio between the *z*-average hydrodynamic diameters of complexes and PCPP samples (50 mM phosphate buffer, pH 7.4, 2 mg mL^−1^ PCPP, ambient temperature). Dense protein-polymer aggregates formed by short-chain PCPP and loose agglomerates of long-chain PCPP are schematically shown.

The formation of protein-polymer complexes was studied by monitoring their dimensions and aggregation patterns using the dynamic light scattering (DLS) titration method. Fig. 2 shows changes in the *z*-average hydrodynamic diameters of PCPP samples of variable molar masses upon the addition of lysozyme. DLS results display a rapid and dramatic increase in the complex size upon titration of short-chain PCPP samples. As the molar mass of PCPP rises, it takes a gradually higher amount of lysozyme to cause detectable changes in the DLS data. Concurrently, the slope of titration curves becomes significantly less steep, indicating a lower trend of aggregation in the case of high-molar-mass polymers (Fig. 2). This suggests the formation of complexes with higher water contents and less compact morphologies – agglomerates, rather than aggregates. It is generally expected that polymers of a sufficient length realize their binding capability by wrapping and winding around proteins and particles.^43–46^ This imminently results in the formation of macromolecular conformations containing multiple loops and tails or gel-like structures^47^ (Fig. 2, right side). In contrast, macromolecules, which are too short to provide for such loose irregularities, are more likely to act as crosslinkers connecting proteins in a much denser, aggregated network^43,46–49^ (Fig. 2, left side). The latter process results in significantly greater entropic penalties compared to the formation of looser assemblies of longer polymer chains. The higher charge density in aggregated systems may also result in an improved retention of counterions, which can contribute to the unfavorable entropy changes observed in the case of short polymer chains.^50^

### 3.4. Complexes formed by shorter chains show reduced accessibility of the protein

Further analysis of complexes involved the assessment of protein accessibility using antibody binding experiments. Potential differences in the detection of polymer-bound lysozymes by the enzyme-linked immunosorbent assay (ELISA) may indicate variations in the morphology of complexes and either support or undermine the importance of results revealed by DLS and ITC studies. The ability of the complex to provide efficient exposure of protein is also important from a practical standpoint. The vaccine delivery function of polyphosphazene immunoadjuvants is largely dependent on the ability of the polymer to make the antigenic protein “visible” to the immune system.

**Fig. 3:**
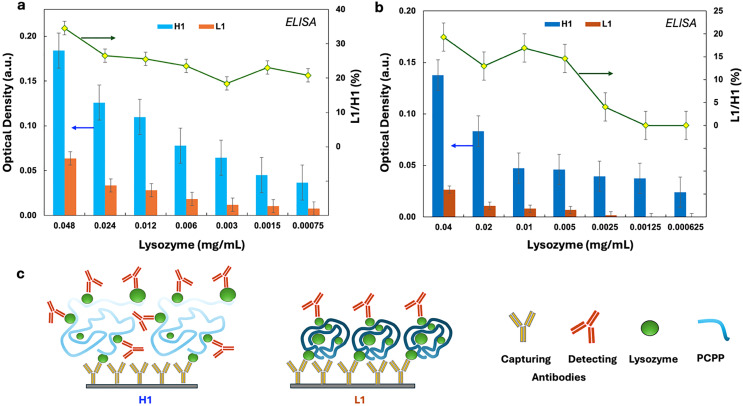
Accessibility of lysozyme in its complexes with high-molar-mass (H1) and low-molar-mass PCPP (L1), as evaluated by ELISA using lysozyme antibodies at (a) 1.5 : 1 and (b) 2.5 : 1 (mmol eq.^−1^) protein-to-carboxylic acid group ratios and (c) schematic of the potential effect of complex morphology on ELISA results (experimental results are shown as optical densities at 450 nm (brown columns – complexes formed by L1, blue – by H1, left axes) or their ratios expressed in percentages (green lines, right axes), *n* = 3, error bars-standard error).

Therefore, the accessibility of proteins in the complexes formed by L1 and H1 polymers was evaluated based on the ability of the complex-bound lysozyme to interact with both capturing and detecting antibodies. Fig. 3a and b show the ELISA assay response at serial dilutions for complexes formed by L1 (brown columns) and H1 (blue columns) at two different protein-to-polymer ratios. The results demonstrate striking differences between the L1- and H1-derived complexes of both compositions and at all concentrations. The ability of antibodies to detect lysozyme bound to shorter chains is greatly suppressed compared to complexes formed by their longer counterparts. Even under the most favorable conditions, the fraction of antibody-recognized proteins in L1-formed complexes does not exceed 30 percent of that detected in H1-derived samples (green lines and diamond symbols in Fig. 3a and b). These findings are in line with the hypothesis that the compact morphology of complex aggregates formed by short-chain PCPP is responsible for the effects observed in DLS and ITC studies. In fact, a loose conformation of a polymer chain should facilitate the access of antibodies to lysozyme, as schematically shown in Fig. 3c.

### 3.5. Larger PCPP macromolecules provide for enhanced stimulation of engineered immune cells

Another important aspect of biological activity inherent to polyphosphazene polyelectrolytes is their ability to interact with the cellular surface. It has been shown that various PCPP copolymers facilitate the uptake of protein cargo into endothelial and cancer cells *via* multiple endocytic pathways.^51^ Intrinsic to the immunostimulating properties of PCPP and its structural analogs is their ability to stimulate immune cells, such as human dendritic cells,^52^ or induce the production of cytokines in mouse spleen cells.^53^

**Fig. 4:**
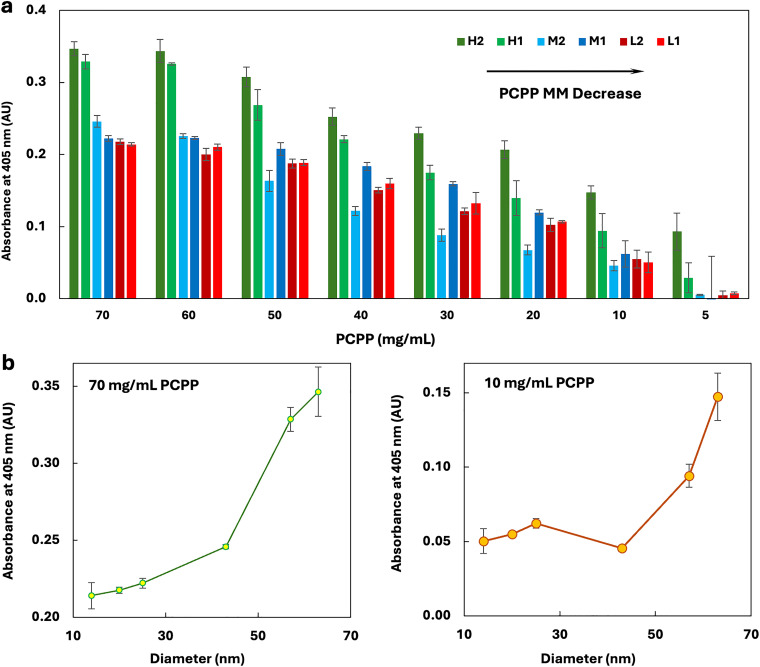
Immunoactivation of RAW BLUE cells by PCPP samples of various molar masses, as measured by the spectrophotometric monitoring of SEAP substrate hydrolysis: experimental results presented as a function of the (a) polymer concentration and (b) *z*-average hydrodynamic diameter of PCPP in PBS, pH 7.4 (405 nm, *n* = 3, error bars-standard error).

Engineered immune cells expressing pattern recognition receptors (PRRs) present a convenient model for studying PCPP-cell interactions. It has been demonstrated that RAW BLUE cells, which are derived from murine macrophages, can be effectively stimulated by PCPP.^35^ Furthermore, the secreted embryonic alkaline phosphatase (SEAP) reporter construct harbored in such cells enables UV-Vis readouts, allowing the straightforward evaluation of the effect of various polymer-related parameters. Importantly, PCPP-induced cell activation has been associated with the ability of PCPP to bind solubilized membrane PRRs, suggesting the potential role of surface proteins in facilitating polymer–cell interactions.^35^ The *in vitro* assessment of the ability of PCPP samples with varying molar masses to stimulate RAW BLUE cells demonstrates the striking effect of the polymer chain length (Fig. 4). An increase in the molar mass of PCPP samples leads to a gradually stronger immunostimulating activity. Although this phenomenon requires further in-depth investigations using other cell types and environmental conditions, the results uncover similar trends to those observed in studying polymer protein self-assembly – the larger polymer size is favored. These findings underline the importance of a systematic review of polymer-induced cellular activation in the context of polymer binding to surface proteins and receptors.

## 4. Conclusion

Preclinical and clinical advancement of synthetic polyelectrolytes has seen significant acceleration in recent years. This progress is largely associated with important innovations in the area of polyelectrolyte self-assembly, with applications ranging from biocompatible multilayer coatings to engineered cells. In the field of drug and vaccine delivery, the evolution of synthetic biodegradable polyelectrolytes with benign degradation byproducts opens a potential pathway to their safe clinical use. The water-soluble polyphosphazene polyelectrolyte, PCPP, with a clinically proven safety and immunoadjuvant potency, is one of the most promising macromolecules of this generation. Mechanistically, PCPP functions *via* its self-assembly with an immunogen and its effective presentation to the immune system. One of the unexplained and intriguing features of its behavior remains the rapid loss of its *in vivo* activity, which is experimentally observed when its molar mass decreases below the 100 kDa threshold. The adverse impact of this property not only limits the utilization of low-molar-mass polymers in clinical settings but also results in a shortened activity window as the hydrolytic degradation of the polymer leads to a simultaneous loss in its potency.

The above *in vitro* findings shift the spotlight from focusing entirely on the stability of PCPP complexes with the protein to emphasizing the morphological aspects of the complex formed and the accessibility of the antigenic protein embedded in its agglomerated matter. Longer polymer chains appear to create additional benefits for the application by creating looser and more accessible assemblies, potentially resulting in a more effective antigen presentation *in vivo*. Perhaps such agglomerated matter may also be more easily recognized by immune cells, as follows from the results of *in vitro* cellular assays. Regardless of the exact mechanism, results suggest the importance of creating looser complex morphologies to enhance the antigen presentation feature of the complex and its cellular recognition. Therefore, the rational approach to the structural design of a new generation of polyphosphazene adjuvants calls for the inclusion of hydrophilic moieties so that the formation of compact aggregates under *in vivo* conditions can be avoided. Although this study was focused on a single example of a synthetic polyelectrolyte, it can be envisioned that similar aspects should be explored when other ionic macromolecules undergo exposure to protein and cellular environments while carrying out vaccine or drug delivery functions.

## Author contributions

Conceptualization: A. K. A.; investigation: R. H., A. M., and A. C.; data curation, formal analysis: R. H., A. M. and A. C.; writing – original draft preparation, writing – review and editing, supervision, project administration, funding acquisition: A. K. A.

## Conflicts of interest

Alexander K. Andrianov is a shareholder of NeuImmune, Inc. – the company that develops HCV vaccine. The company had no role in the design of the study; collection, analysis, or interpretation of the data; writing of the manuscript; or the decision to publish the results. This potential conflict of interest has been disclosed and is managed by the University of Maryland, College Park. The other authors have no conflicts of interest to report.

## Supplementary Material

TB-014-D5TB02163D-s001

## Data Availability

The data supporting this article have been included as part of the supplementary information (SI). Supplementary information: dynamic light scattering profiles, size exclusion chromatograms of polymers, isothermal titration calorimetry results. See DOI: https://doi.org/10.1039/d5tb02163d.
