# Supplementary material for: Macromolecular dimensions of a synthetic polyelectrolyte as a factor in its interactions with protein and cells: desirability for longer chains
Source: J Mater Chem B. 2025 Nov 27;14(2):563–71. doi: 10.1039/d5tb02163d (PMC12704047; doi:10.1039/d5tb02163d)
Supplement: TB-014-D5TB02163D-s001 [file TB-014-D5TB02163D-s001.pdf]

Supplementary Information (SI) for Journal of Materials Chemistry B.
This journal is © The Royal Society of Chemistry 2025

Macromolecular Dimensions of a Synthetic Polyelectrolyte as a Factor in its Interactions with Protein and Cells – Longer Chains are Favoured

*Raman Hlushko, Alexander Marin, Ananda Chowdhury[#] and Alexander K. Andrianov**

Institute for Bioscience and Biotechnology Research, University of Maryland, Rockville, MD 20850, USA

[#]Present address: PREMISE, Vaccine Research Center, National Institute of Allergy and Infectious Diseases, National Institutes of Health, Gaithersburg MD, 20878, USA

*Correspondence: aandrianov@umd.edu (A.K.A)

Supplementary Information

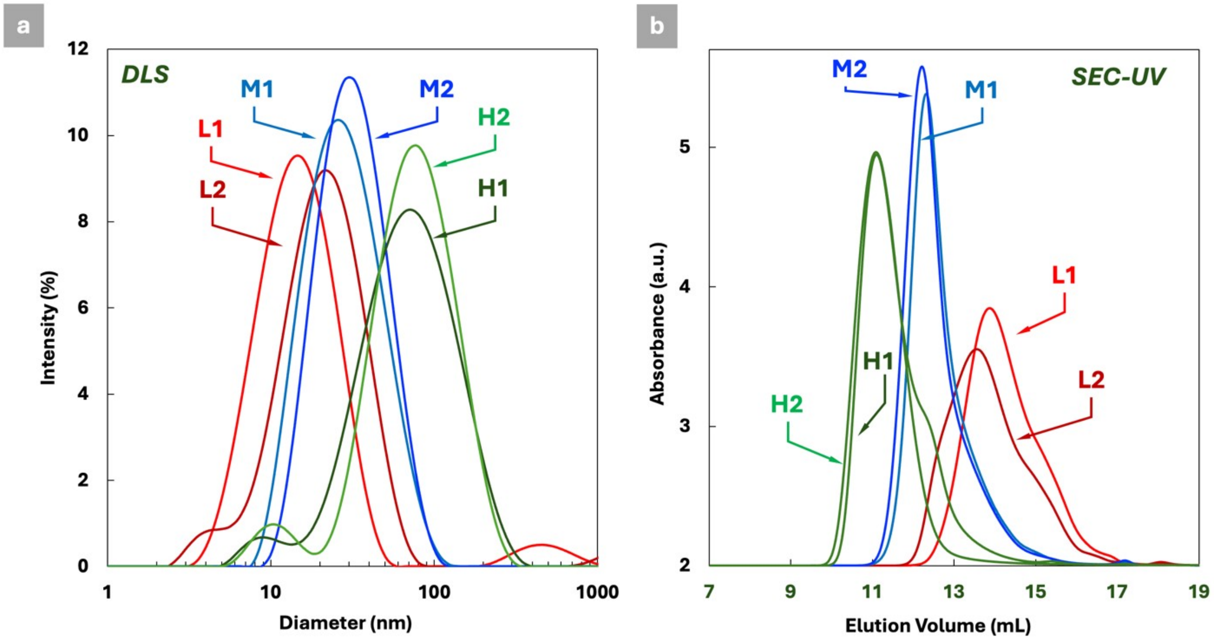

Figure S1. (a) Dynamic light scattering (DLS) profiles and (b) size exclusion chromatograms (SEC) of polymers (1 mg/mL PCPP, PBS, pH 7.4, SEC: UV detection at 210 nm)

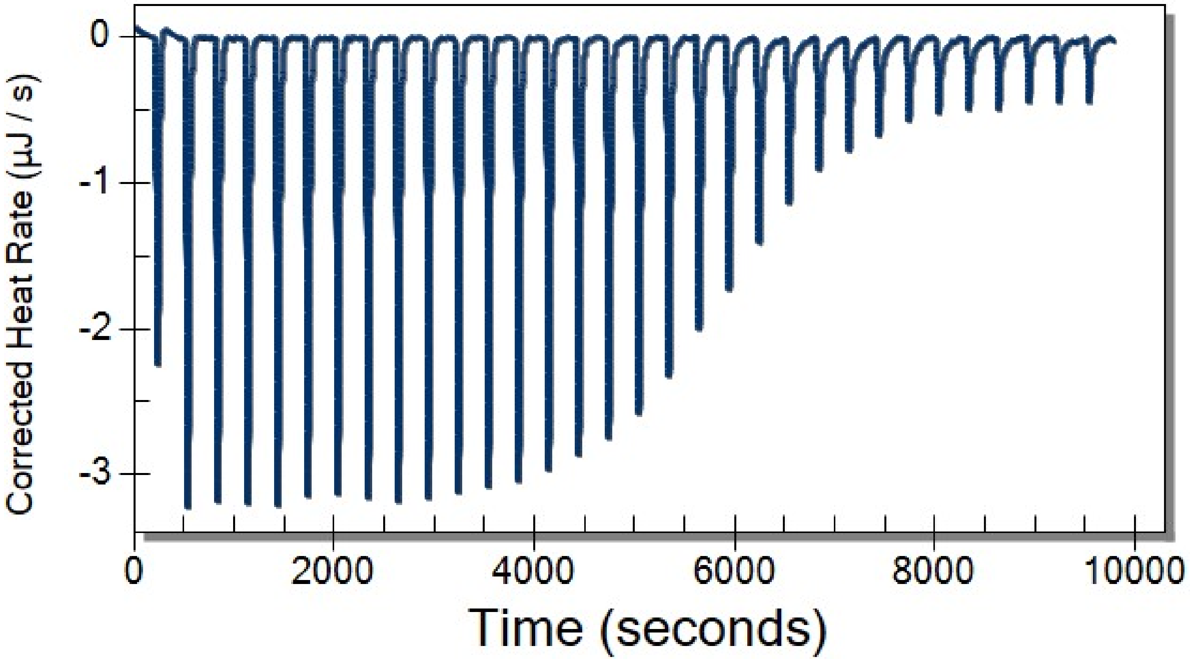

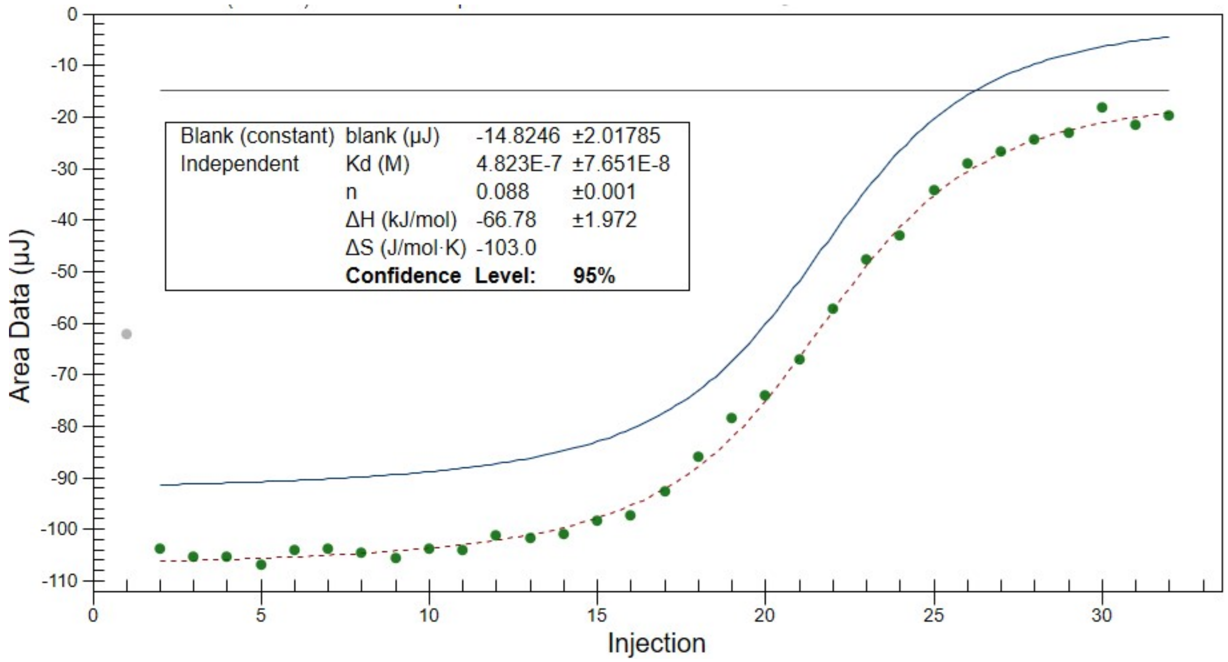

Fig. S2. ITC titration of PCPP-L1 with lysozyme (0.125 mg/mL polymer, 2.5 mg/mL protein, 50 mM phosphate buffer, pH 7.5).

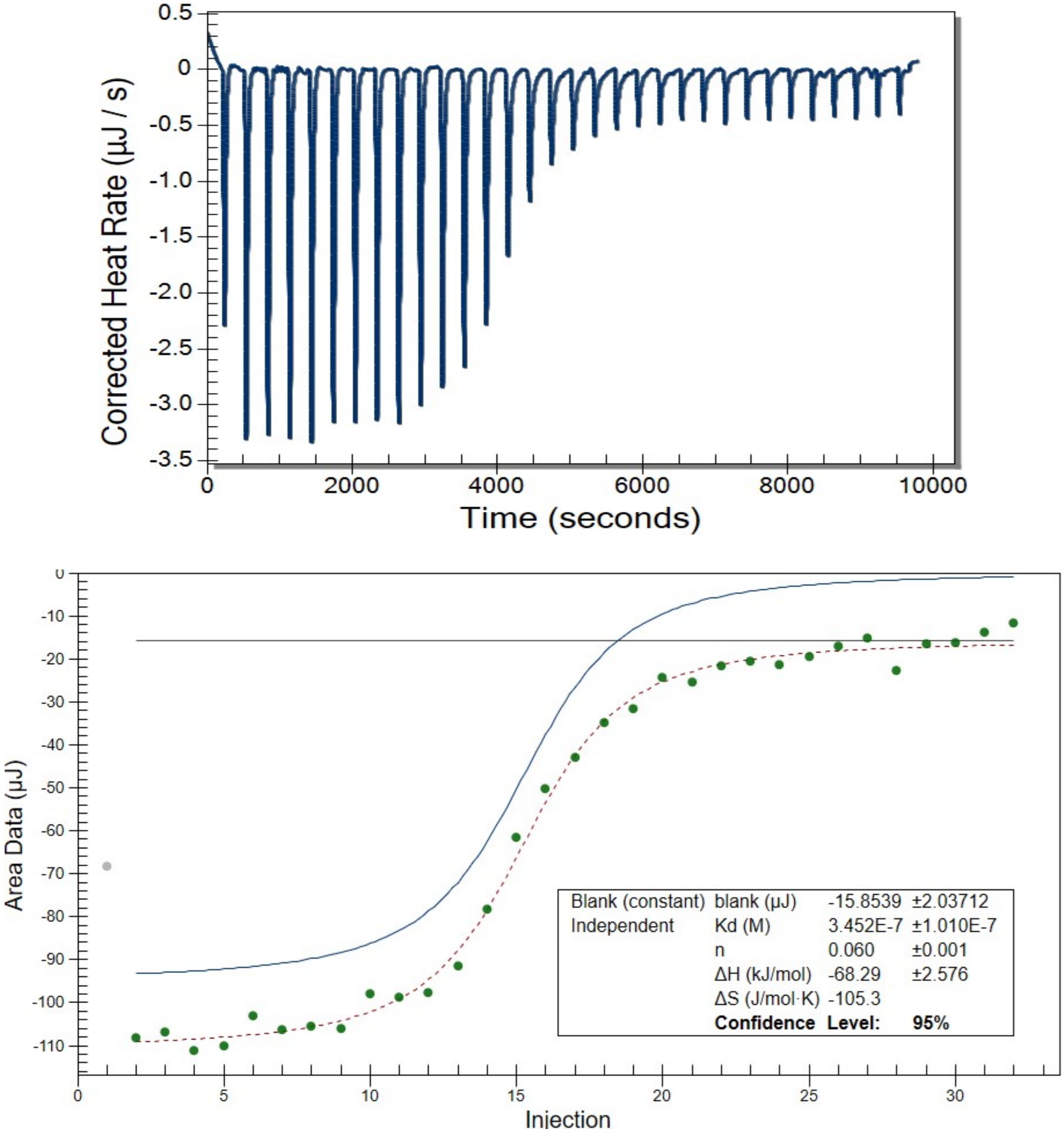

Fig. S3. ITC titration of PCPP-L2 with lysozyme (0.125 mg/mL polymer, 2.5 mg/mL protein, 50 mM phosphate buffer, pH 7.5).

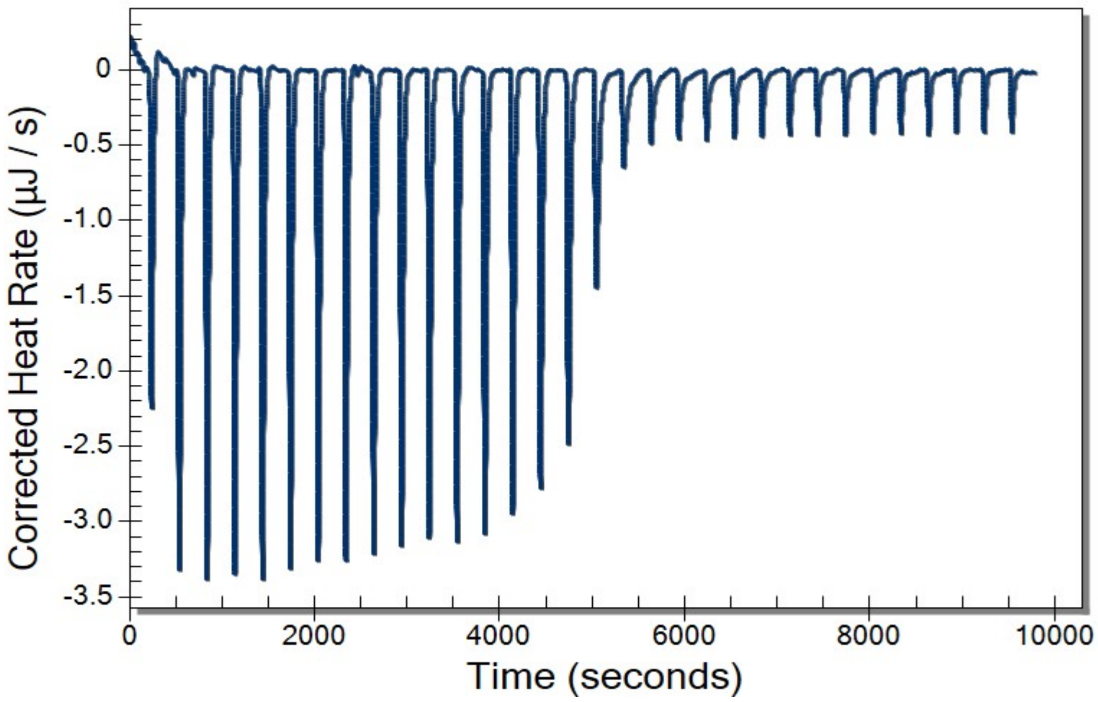

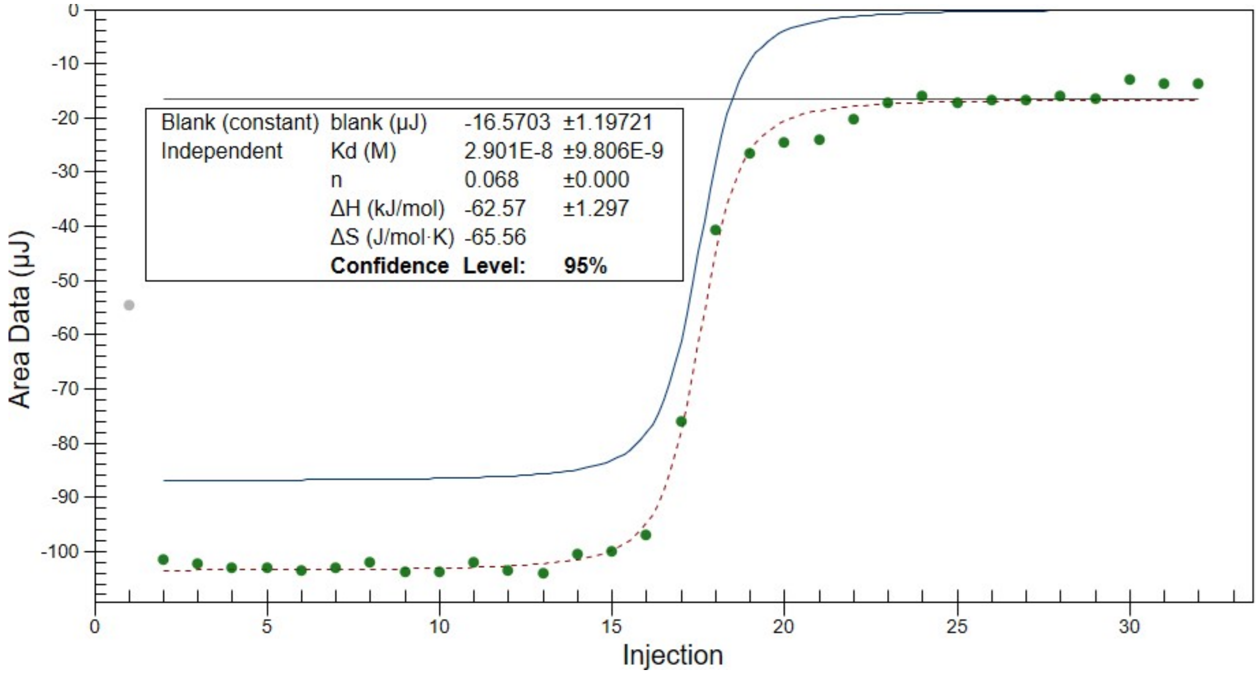

Fig. S4. ITC titration of PCPP-M1 with lysozyme (0.125 mg/mL polymer, 2.5 mg/mL protein, 50 mM phosphate buffer, pH 7.5).

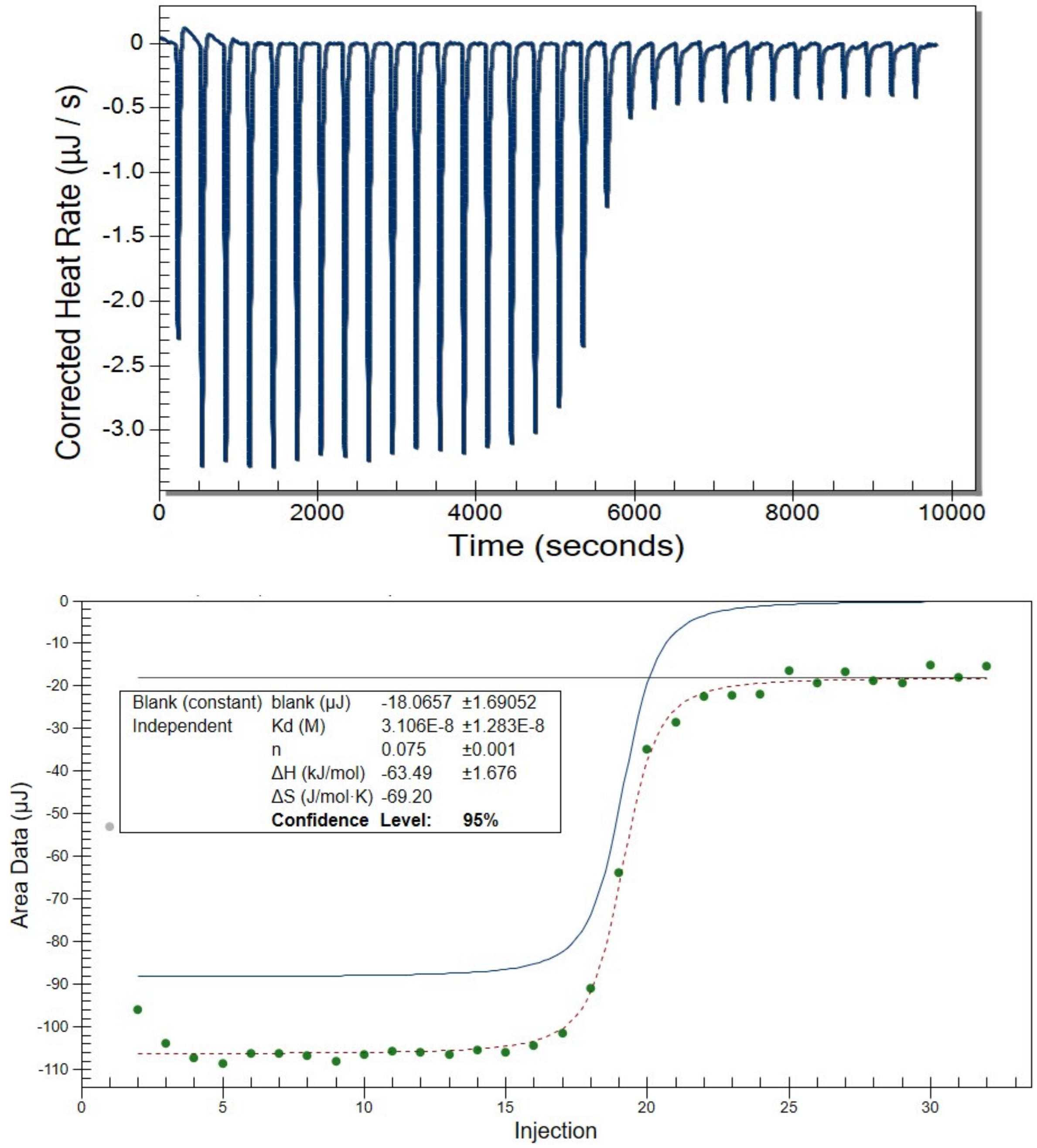

Fig. S5. ITC titration of PCPP-M2 with lysozyme (0.125 mg/mL polymer, 2.5 mg/mL protein, 50 mM phosphate buffer, pH 7.5).

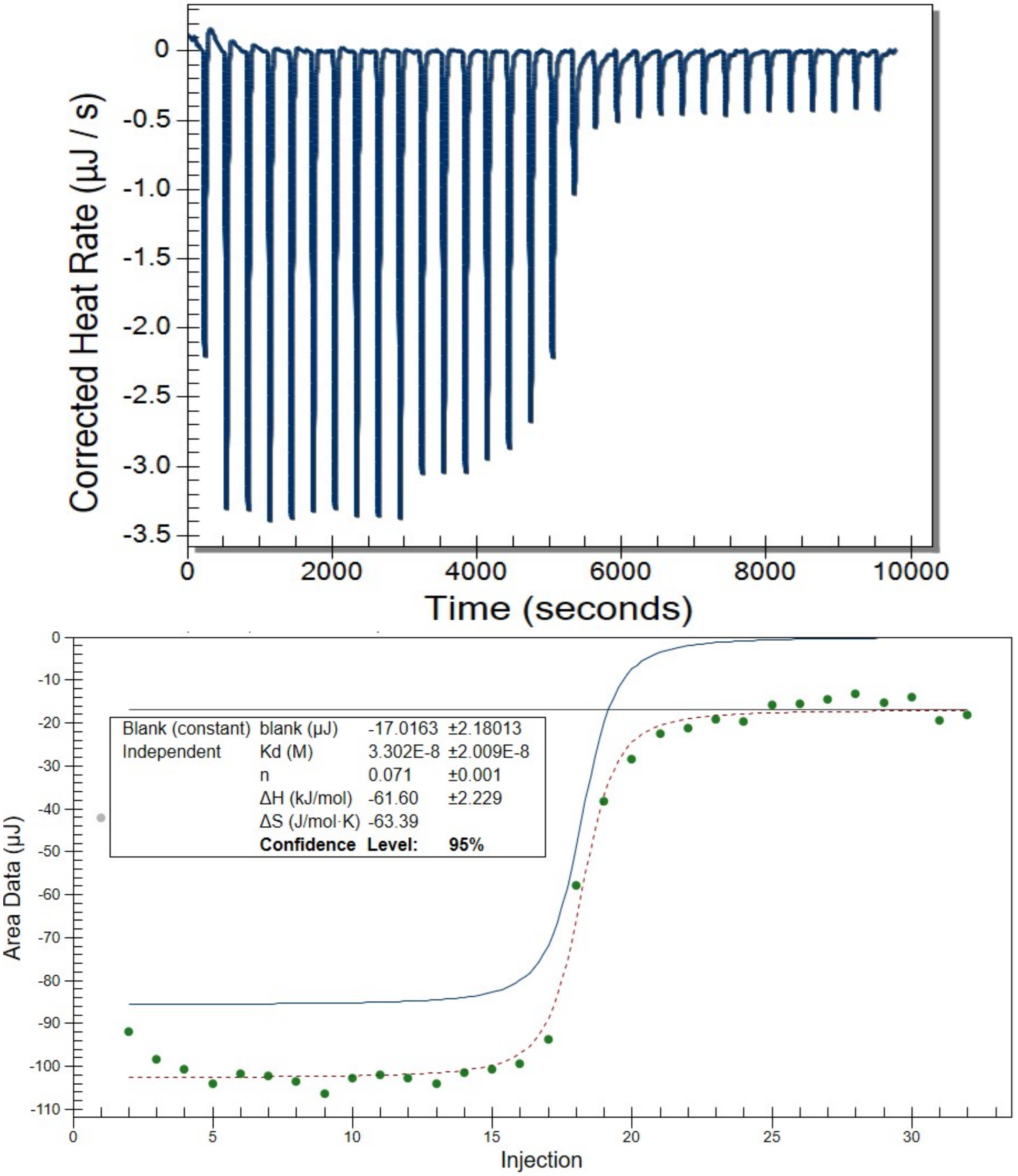

Fig. S6. ITC titration of PCPP-H1 with lysozyme (0.125 mg/mL polymer, 2.5 mg/mL protein, 50 mM phosphate buffer, pH 7.5).

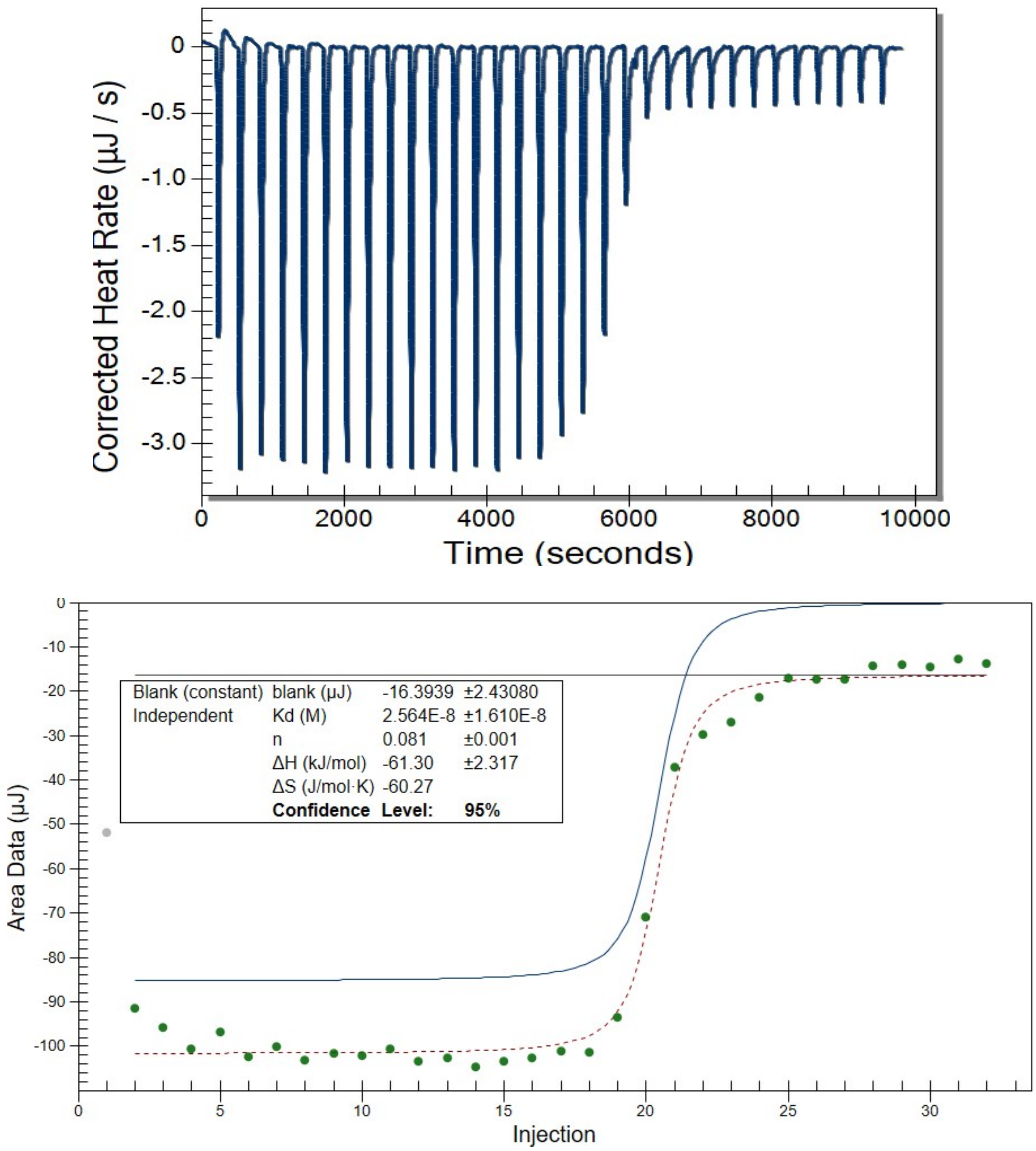

Fig. S7. ITC titration of PCPP-H2 with lysozyme (0.125 mg/mL polymer, 2.5 mg/mL protein, 50 mM phosphate buffer, pH 7.5).
